# Blood–Brain Barrier Permeability Study of Potential Neuroprotective Compounds Recovered From Plants and Agri-Food by-Products

**DOI:** 10.3389/fnut.2022.924596

**Published:** 2022-06-16

**Authors:** José David Sánchez-Martínez, Alberto Valdés, Rocio Gallego, Zully Jimena Suárez-Montenegro, Marina Alarcón, Elena Ibañez, Gerardo Alvarez-Rivera, Alejandro Cifuentes

**Affiliations:** ^1^Laboratory of Foodomics, Institute of Food Science Research, CIAL, Spanish National Research Council (CSIC) - Universidad Autónoma de Madrid (UAM), Madrid, Spain; ^2^Area of Food Technology, Faculty of Chemical Sciences and Technologies, University of Castilla-La Mancha, Ciudad Real, Spain

**Keywords:** neuroprotective, blood-brain barrier, green extraction, UHPLC-Q-TOF-MS, bioactive compounds, agri-food by-products

## Abstract

Plants and agri-food by-products represent a wide and renewable source of bioactive compounds with neuroprotective properties. In this research, various green extraction techniques were employed to recover bioactive molecules from *Kalanchoe daigremontiana* (kalanchoe), epicarp of *Cyphomandra betacea* (tamarillo), and cooperage woods from *Robinia pseudoacacia* (acacia) and *Nothofagus pumilio* (lenga), as well as a reference extract (positive control) from *Rosmarinus officinalis L*. (rosemary). The neuroprotective capacity of these plant extracts was evaluated in a set of *in vitro* assays, including enzymatic [acetylcholinesterase (AChE), butyrylcholinesterase (BChE), and lipoxygenase (LOX)] and antioxidant [ABTS, and reactive oxygen and nitrogen species (ROS and RNS)] bioactivity tests. Extracts were also submitted to a parallel artificial membrane permeability assay mimicking the blood–brain barrier (PAMPA-BBB) and to two cell viability assays in HK-2 and SH-SY5Y cell lines. Comprehensive phytochemical profiling based on liquid chromatography coupled to quadrupole-time-of-flight mass spectrometry (LC-Q-TOF-MS) analysis showed enriched content of phenolic and terpenoid compounds in the target extracts. Moreover, *in vitro* bioactivity tests showed promising neuroprotective capacity, particularly for supercritical-fluid extraction (SFE) extract from acacia (ABTS IC_50_ = 0.11 μg ml^−1^; ROS IC_50_ = 1.56 μg ml^−1^; AChE IC_50_ = 4.23 μg ml^−1^; BChE IC_50_ = 1.20 μg ml^−1^; and LOX IC_50_ = 4.37 μg ml^−1^), whereas PAMPA-BBB assays revealed high perfusion capacity of some representative compounds, such as phenolic acids or flavonoids. Regarding cytotoxic assays, tamarillo and rosemary SFE extracts can be considered as non-toxic, acacia SFE extract and lenga pressurized liquid extraction (PLE) extract as mild-cytotoxic, and kalanchoe as highly toxic extracts. The obtained results demonstrate the great potential of the studied biomass extracts to be transformed into valuable food additives, food supplements, or nutraceuticals with promising neuroprotective properties.

## Introduction

Agricultural and food industries generate tons of waste that have led to environmental and economic issues. However, these residues can be considered as renewable and low-cost sources of natural compounds with potential bioactivity (1). In this sense, the valorization of agri-food by-products like seeds, leaves, peels, weeds, and woods using green extraction approaches, such as pressurized liquid extraction (PLE), supercritical-fluid extraction (SFE), and ultrasound-assisted extraction (UAE), represents an ideal opportunity to recover bioactive molecules that can be delivered into the market as new functional foods or nutraceuticals.

Both PLE and SFE techniques are based on the use of compressed fluids for the effective extraction of bioactive compounds from natural sources. Their high efficiency relies on the use of high temperature and pressure conditions, which changes the physicochemical properties of extraction solvents and improves the solubility of analytes, while decreasing the tension and viscosity of the solvent surface (2). The UAE technique can induce thinning and poration of vegetal membranes, which subsequently increase solvent permeability across the vegetal matrix. UAE has been demonstrated to efficiently extract thermo-labile compounds from food matrices without degradation (2). Neurodegenerative disorders are a group of progressive neurological diseases characterized by cognitive impairment, loss of memory, and anxiety, among other symptoms. Alzheimer's disease (AD) is considered to be the main neurodegenerative disorder affecting 50 million people in 2018, and the number of cases can be increased to 152 million by 2050 (3). The neuropathology mechanism of AD is still unclear; however, several findings showed aggregation of amyloid-beta (Aβ) plaques, hyper-phosphorylation of tau proteins and their aggregation into neurofibrillary tangles (NFT), oxidative stress, neuroinflammation, and cholinergic hypothesis as principal hallmarks of AD (4). The cholinergic hypothesis refers to a decline in the production of acetylcholine (ACh) neurotransmitters in the synaptic cleft that is closely related to progressive cognitive impairment in AD patients (5). In fact, nowadays there is no effective cure for AD, and the palliative treatment is based on N-methyl-D-aspartate receptor antagonist and acetylcholinesterase (AChE) and butyrylcholinesterase (BChE) inhibitors (6, 7). Moreover, AChE and BChE are associated with an increase in the formation of Aβ plaques (4). Likewise, chronic neuroinflammation in AD is related to Aβ plaque aggregation and lipoxygenase (LOX) enzymatic activity, both mechanisms promoting the synthesis of pro-inflammatory response mediators (cytokines, leukotrienes, and chemokines among other mediators) (8). Meanwhile, oxidative stress damage in AD patients is caused by free radicals, such as reactive oxygen species (ROS) and reactive nitrogen species (RNS), which are also induced by Aβ plaque aggregation and pro-inflammatory response mediators that can lead to neuronal cell death *via* mitochondrial alterations (8, 9). In consequence, cell death also promotes the free radical generation and a pro-inflammatory scenario by an immune response that supports Aβ plaque and NFT accumulation. All these conditions lead to a feedback loop between neuroinflammation, oxidative stress, and neurodegeneration in AD. For this reason, over the decades, several researchers focused their efforts on identifying and extracting neuroprotective compounds from natural sources like plants (10, 11). In fact, terpenoids, phenolic compounds, and alkaloids, among other natural compounds, exhibit anti-inflammatory, anticholinergic, and antioxidant capacity, and also provide protection against Aβ plaque formation, as reported from *in vivo* and *in vitro* experiments (12, 13).

In spite of the promising neuroprotective properties reported for some natural compounds, it is crucial to demonstrate their permeability across the blood–brain barrier (BBB) to guarantee that they can reach brain tissues and exert the claimed neuroprotective capacity. In fact, nearly 100% of large molecules and around 98% of small neurotherapeutic molecules are not capable of crossing the BBB (14). In this line, various *in silico, in vitro*, and *in vivo* models have been developed to study the permeability of bioactive compounds across the BBB. Parallel artificial membrane permeability assay for the blood–brain barrier (PAMPA-BBB) was first used by Di et al. in 2003 (15) and represents a low-cost and high-throughput non-cell-based and reproducible permeability test, which is ideal for novel drugs or therapeutic bioactive compounds (14). Hence, the goal of the present study is to perform a comprehensive chemical characterization by HPLC-Q-TOF-MS/MS and evaluate the neuroprotective properties of a set of natural extracts, involving a succulent plant (*Kalanchoe daigremontiana*) and four agri-food by-products, such as epicarp from *Cyphomandra betacea* (tamarillo), cooperage woods from *Robinia pseudoacacia* (acacia) and *Nothofagus pumilio* (lenga), and a reference extract from *Rosmarinus officinalis L*. (rosemary). To achieve this aim, a set of *in vitro* bioactivity assays, including anticholinesterase enzymatic activity (AChE and BChE), anti-inflammatory enzymatic activity (LOX), and antioxidant capacity (ABTS, ROS, and RNS), as well as *in vitro* cytotoxicity by using different human cell culture models (HK-2 and SH-SY5Y cells), were performed. Finally, *in vitro* permeability of the bioactive compounds isolated from each extract across the BBB is evaluated by the PAMPA-BBB methodology.

## Materials and Methods

### Selection of Natural Biomass and Extraction Conditions of Bioactive Compounds

For this work, a set of plants and agri-food by-products highly enriched in bioactive phytochemicals were selected to test their neuroprotective potential. First, *K. daigremontiana* (kalanchoe) is an herbaceous plant whose roots have been used for decades in traditional medicine due to its anti-inflammatory and anti-tumoral properties. Kalanchoe extracts have also been shown to possess a high content of neuroprotective phenolic compounds (16). Second, epicarp from *C. betacea* (tamarillo) has been described as a valuable source of vitamins, carotenoids, and polyphenols, among others, with reported antioxidant and anti-inflammatory properties (17, 18). Third, cooperage woods, such as those from *R. pseudoacacia* (acacia) and *N. pumilio* (lenga), are commonly used during wine production to impart pleasant aroma and sensorial properties. After their industrial applications, these by-products have been shown to contain high levels of valuable phenolic compounds with high antioxidant capacity (19). Finally, the neuroprotective capacity of *R. officinalis* L. (rosemary) has been demonstrated in previous studies, and a rosemary extract obtained under optimized conditions was used as a “positive control extract and the natural gold standard.”

The extraction of bioactive compounds from these natural biomass extracts was performed under optimized conditions previously developed by our research group. Thus, kalanchoe roots provided by CEPROBI–IPN center (Centro de Desarrollo de Productos Bióticos, Yautepec Morelos, México) were washed with water and dried at room temperature for 10 days. Roots were grounded and stored at room temperature. Ethanolic extracts from kalanchoe roots (KD) were obtained by UAE as previously described by Bravo (2020) (20). Roots were placed in an Eppendorf tube with EtOH/H_2_O (8: 2, v/v), the mixture was sonicated for 30 min at 40°C (BRANSON 2800), and the extract was separated from the raw material by centrifugation (Eppendorf centrifuge 5804R, Hamburg, Germany) at 3,000 rpm for 5 min. Finally, the supernatants were collected, and the solvent was evaporated in a SpeedVac system at 40°C and 0.0013 MPa. The final extracts were stored at −18°C. UAE methodology was employed to improve the extraction of bioactives from kalanchoe roots, a resistant plant tissue that requires an energetic pretreatment. Ultrasound can induce thinning and poration of vegetal membranes, which consequently increases solvent permeability across the vegetal matrix.

Tamarillo yellow ecotype fruit was purchased from a local market in Colombia. Tamarillo peel was removed manually and washed with distilled water and dried at room temperature for 72 h. After that, the peel was grounded, sieved to a particle size between 250 and 500 μm, and vacuum-packed for storage at −18°C. Tamarillo peel extract (T33) was obtained by PLE as previously described by our group (18). In brief, the extraction was performed using a Dionex accelerated solvent extract (ASE 200, Sunnyvale, CA, USA) operating at 10 MPa. The extraction conditions were 100% EtOH, 180°C, and 20 min of extraction time. The extract was then centrifuged (Eppendorf centrifuge 5804R, Hamburg, Germany) at 10,000 rpm for 30 min at 10°C, and the supernatant was collected (S1). Precipitates associated with low EtOH solubility of pectins were washed with EtOH and centrifuged again to obtain a second supernatant (S2) that was mixed with S1, constituting the final T33 extract. Finally, T33 extract was dried under N_2_ and stored at −18 °C. The use of high? temperature and pressure (enough to maintain the solvent in the liquid state) in PLE led to a decrease in solvent surface tension and viscosity, whereas the solubility of analytes increased, ultimately improving the mass transfer rates. Using combinations of polar solvents, such as water and/or EtOH, the phenolic fraction of LPLE and T33 extracts could be successfully obtained.

Unroasted cooperage wood samples of acacia and lenga were kindly provided by Tonelería Magreñán S. L. (La Rioja, España). These wood samples were grounded using mechanical milling and further sieved (particle size between 500 and 1,000 μm) to obtain homogenous sawdust that was stored at room temperature. Acacia extracts (ASFE) were obtained by SFE under the following conditions: 60°C in the extraction cell, 23.5 MPa as extraction pressure, and 45% of co-solvent [EtOH/H_2_O (1: 1, v/v)] in 60 min of extraction time. Lenga extract (LPLE) was obtained by a PLE extraction process, using EtOH/H_2_O (1: 1, v/v) as a solvent at 120°C in two 10-min static cycles operating at 10 MPa. Both the extraction processes of LPLE and ASFE were previously optimized by Alarcon (21). After extraction, the solvent was removed under N_2_ flow at 40°C and then freeze-dried (Lyobeta, Telstar, Terrassa, Spain).

Rosemary leaves were acquired from Herboristeria Murciana (Murcia, Spain). Rosemary leaves were dried in a ventilated place for 20–30 days at room temperature and covered from the direct light. Then, cryogenic sample grinding was performed under liquid nitrogen and sieved to a particle size between 500 and 1,000 μm. The leaf samples were stored at −18°C. Rosemary extract (RSFE) was obtained through a pilot-scale SFE (Thar Technology, Pittsburgh, PA, USA, model SF2000) as previously described by our group (22). In brief, the temperature in the SFE extraction cell was set at 40 °C, and the CO_2_ flow rate was maintained at 60 g/min for 5 h. The extraction pressure was 15 MPa, and 7% ethanol was used as a co-solvent modifier. Once the extract was collected, a Rotavapor R-210 (from Büchi Labortechnik AG, Flawil, Switzerland) was used for solvent evaporation, and the extracts were kept at −18°C. SFE with CO_2_ provides a green extraction alternative for low-polarity molecules. The addition of co-solvents like EtOH allows the widening of the polarity range of extractable analytes. Thus, SFE was selected to obtain diterpenoids with a high added value (carnosic acid and carnosol) in RSFE extracts, whereas a wider range of polarities was covered in ASFE extracts by using CO_2_ with polar co-solvents like EtOH and H_2_O.

### Reagents and Materials

The HPLC-grade acetonitrile and ethanol (EtOH) solvents were acquired from VWR Chemicals (Barcelona, Spain). Acetylcholinesterase (AChE) Type VI-S from *Electrophorus electricus*, butyrylcholinesterase from equine serum (BChE), acetylthiocholine iodide (ATCI), 2,2′-azino-bis(3-ethylbenzothiazoline-6-sulphonic acid) diammonium salt (ABTS), sodium carbonate (Na_2_CO_3_), potassium persulfate, fluorescein sodium salt, sulphanilamide, naphthylethylene diamine dihydrochloride, phosphoric acid, formic acid, gallic acid, ascorbic acid, quercetin, cholesterol, Trizma hydrochloride (Tris-HCl), disodium phosphate (Na_2_HPO_4_), linoleic acid (LA), 2,2'-azino-bis (3-ethylbenzothiazoline-6-sulphonic acid) monopotassium phosphate (KH_2_PO_4_), sodium nitroprusside dehydrate (SNP), n-dodecane, porcine polar brain lipid (PBL), PAMPA-BBB 96-well-donor plate (Catalog No MAIPNTR10), and 96-well-acceptor plate (Catalog No MATRNPS50) were purchased from Sigma-Aldrich (Madrid, Spain). Galantamine hydrobromide, lypoxidase from glycine max (soybean), 4-(amino-359 sulfonyl)-7-fluoro-2,1,3-benzoxadiazole (ABD-F), 2,2-azobis(2-amidinopropane) dihydrochloride (AAPH), and 3-(4,5-dimethylthiazol-2-yl)-2,5-diphenyltetrazolium bromide (MTT) were obtained from TCI Chemicals (Tokyo, Japan). Ultrapure water was obtained by using a Millipore system (Billerica, MA, USA). The human proximal tubular epithelial cells (HK-2) and SH-SY5Y neuroblastoma cell lines were acquired from ATCC^®^ (Rockville, MD, USA.). Cell culture medium Dulbecco's Modified Eagle's Medium Nutrient Mixture (DMEM/Ham's F12), fetal bovine serum (FBS), L-glutamine, antibiotic-antimycotic solution (including streptomycin, penicillin, and amphotericin B), and insulin-transferrin-selenium (ITS) were obtained from Thermo Fisher (Grand Island, NY, USA). Retinoic acid (RA) was acquired from Glentham Life Science (Corsham, United Kingdom), and brain-derived neurotrophic factor (BDNF) was acquired from Bachem AG (Bubendorf, Switzerland). All 96-well-microplate assays were performed in a spectrophotometer with a fluorescence reader (Synergy HT,BioTek Instruments, Winooski, VT, USA).

### Chemical Characterization by Liquid Chromatography Quadrupole-Time-of-Flight Mass Spectrometry (UHPLC-Q-TOF-MS) Analysis

Phytochemical profiling of the studied plant extracts was performed by liquid chromatography (Agilent 1290 UHPLC system) coupled to a quadrupole-time-of-flight mass spectrometer (Agilent 6540 Q-TOF MS) equipped with an orthogonal ESI source. A Zorbax Eclipse Plus C18 column (2.1 × 100 mm, 1.8 m particle diameter, Agilent Technologies, Santa Clara, CA) at 30°C was used. For chromatographic separation, a 5-μl aliquot of each extracted sample was injected at a 0.5 ml/min flow rate. The mobile phase was composed of water (0.01% formic acid) as solvent A and acetonitrile (0.01% formic acid) as solvent B. The gradient elution program was as follows: 0 min, 0% B; 7 min, 30% B; 9 min, 80% B; 11 min, 100% B; 13 min, 100% B; and 14 min, 0% B. Each extracted sample was injected in duplicate.

The mass spectrometer was operated in MS and MS/MS modes. Selected MS parameters were as follows: 10 L/min drying in gas flow rate, 4,000 V as capillary voltage, nebulizer pressure was 40 psi, 350°C as gas temperature, skimmer voltage was 45 V, and fragmentor voltage was 110 V. MS and auto MS/MS modes were used at a scan rate of 5 spectra per second, and m/z values were acquired in the range between 50 and 1,100 and 50–800, respectively. Data processing was carried out using the Agilent MassHunter software package (B.07.00) to obtain qualitative and quantitative information on the tentatively identified compounds. Filtering tools based on diagnostic product ions and/or neutral losses of interest, together with a search in MS/MS databases (NIST, HMDB, Metlin), were useful structural elucidation strategies that greatly enhanced data interpretation. Additional information about each bioactive compound, such as LogP (octanol-water partition coefficient) or topological polar surface area (TPSA), was obtained from PubChem database (https://pubchem.ncbi.nlm.nih.gov/). ClassyFire Batch by Fiehn Lab software was used for the structural classification of compounds into their respective families (23).

### *In vitro* Bioactivity Assays to Determine Neuroprotective Capacity

The neuroprotective capacity of the studied extracts was evaluated through a set of *in vitro* tests. Antioxidant activity assays against ABTS^∙+^, ROS, and RNS free radicals, and enzymatic inhibition activity based on AChE, BChE, and LOX fluorescent methodologies were performed as previously reported by our laboratory (24). The positive control for antioxidant assays was ascorbic acid, while quercetin and galantamine were used as a positive control for LOX and cholinesterase (AChE and BChE) enzymes, respectively.

### PAMPA-BBB Assay to Determine *in vitro* BBB Permeability

The PAMPA-BBB assay was conducted according to the methodology followed by Di et al., with some modifications as described below (15). First, the BBB solution was prepared by dissolving 8 mg of PBL and 4 mg of cholesterol in 600 μl of n-dodecane. The initial donor solution was prepared by mixing 1 ml of extract (10 mg ml^−1^ in EtOH) with 1 ml of buffer (PBS, pH = 7.4, 10 mM). The filter membrane of the donor plate was carefully coated with 5 μl of BBB solution. The acceptor plate was filled with 350 μl of buffer, and the donor plate was carefully assembled over the acceptor plate like a “sandwich.” Finally, the donor plate was filled with 350 μl of donor solution, covered, and incubated for 4 h at 37°C in the dark. After incubation time, 300 μl of the solution was taken from both plates, transferred to separate vials, and dried in the SpeedVac system at 40°C and 13 mbar pressure. Dried acceptor and donor solution were dissolved in 50 μl of pure EtOH before subjecting to UHPLC-Q-TOF-MS analysis. Permeability across *in vitro* BBB of the bioactive compound was measured according to Equation (1) (25), with some modifications in concentration rates.


(1) $$\begin{array}{l} P_{e}=\frac{-\ln\begin{array}{c} \left[1-\frac{C_{A}\left(t\right)}{C_{e}}\right] \end{array}}{A^{*}{\left(\frac{1}{V_{D}}+\frac{1}{V_{A}}\right)}^{*}t}\; \end{array}$$

where P_e_ refers to the permeability of bioactive compound across PAMPA-BBB in cm s^−1^, A is the effective filter area = 0.3 cm^2^ (given by manufacturer), V_D_ is the donor well volume = 0.35 ml, V_A_ is the acceptor well volume = 0.35 ml, t is the incubation time (s) = 14,400, C_A_(t) is the relative area of the compound in acceptor well-at time t, and C_D(t)_ is the relative area of the compound in donor well-at time t. C_equilibrium_ was calculated according to the following equation (2):


(2) $$\begin{array}{l} C_{equilibrium}=\left[C_{D\left(t\right)}^{*}V_{D}+C_{A\left(t\right)}^{*}V_{A}\right]/\left(V_{D}+V_{A}\right) \end{array}$$

### Cell Culture Growth Conditions and *in vitro* Cytotoxicity Assay

The *in vitro* cytotoxicity of the plant extracts was evaluated using two different human cell culture lines: human proximal tubular epithelial cells (HK-2) and human neuroblastoma cells (SH-SY5Y). HK-2 cells were cultured as previously described by Suárez Montenegro et al. (26). HK-2 cells were grown in 96-well-plates at a density of 5 × 10^3^ cells/well. After 24 h, cells were exposed to different concentrations of plant extracts and were incubated for 24 h. MTT assay was used to determine the viability of HK-2 cells (27). In short, after 24 h of extract exposition, the cell culture medium was removed, and then cells were incubated with 0.5 mg ml^−1^ of MTT solution for 3 h at 37°C. After that, DMSO solvent was used to solubilize the formazan crystals, and then absorbance was reordered at 570 nm in a microplate reader.

The SH-SY5Y cells were grown and differentiated by the method previously proposed by Medeiros et al. in 2019 (28). Thereafter, differentiated cells were grown in 24-well-plates at a density of 4.2 × 10^4^ cells/cm^2^ for 24 h. For the *in vitro* neurotoxic evaluation of plant extracts, cells were exposed to different concentrations for 24 h, and an MTT assay was used to assess the viability of the cells. In both cell lines, the cytotoxicity of the different concentrations of plant extracts is expressed as relative cell viability, which is shown as the percentage of living cells compared to controls (EtOH-treated). In all cases, EtOH was used as extract solvent and did not exceed 0.4% (v/v) in the cell medium.

### Statistical Analysis

All *in vitro* experiments were performed in three independent assays. IC_50_ values given in μg ml^−1^ were calculated by the ID% of each extracted sample that was plotted at seven different concentrations in order to obtain concentration-dependent curves by linear regression (Microsoft excel 2010, Washington USA). All experimental results are shown as mean ± standard deviation (Mean ± SD). ANOVA test was used to analyze the mean values from experimental data results that were compared by Tukey's HSD tests (SPSS statics V26 IBM, New York, USA). They are indicated by different alphabetical letters along with mean values in the tables. A value of *p* < 0.05 was considered to be statistically significant.

## Results and Discussion

A wide range of natural compounds, such as flavonoids, terpenoids, bufanolides, and phenolic acids, among others, were found in the target plant extracts (RSFE, KD, T33, LPLE, and ASFE) (see Tables 1–**5**). These extracts were submitted to comprehensive chemical characterization, followed by *in vitro* biological activity testing, *in vitro* cytotoxicity, and BBB permeability evaluation assays as discussed in the following sections. RSFE extract (rosemary) was used as a “positive control extract” for comparison purposes, considering its well-known anticholinergic, antioxidant, and anti-inflammatory properties that can be attributed to the presence of major compounds like carnosol and carnosic acid, among others. For this reason, several studies have highlighted the large *in vitro* and *in vivo* neuroprotective effects of rosemary extracts (29, 30).

**Table 1:** Phytochemical profile, abundance (%), and BBB permeability of RSFE (Rosmarinus officinalis L., SFE extract).

**Ret. Time**		**Abundance**		**RSD**	**Molecular**	**[M-H]- (m/z)**			**Family**	**PAMPA-BBB log**		**RSD**	**Cross BBB**
**(min)**	**Compound**	**(%)**		**(%)**	**formula**	**(theoretical)**	**Log P**	**TPSA**	**compound**	**Pe** **(cm s−1)**		**(%)**	**potential**
0.598	Quinic acid	0.13		1.8	C7H12O6	191.0561	−2.4	118	Cyclic alcohols	−5.22		7.1	-
2.489	Protocatechuic acid	0.06		1.6	C7H6O4	153.0193	1.1	78	Hydroxybenzoic acids	−3.83		1.9	+++
3.258	p-salycilic acid	2.54		0.7	C7H6O3	137.0244	1.6	58	Hydroxybenzoic acids	−4.18		3.4	+++
3.841	Caffeic acid	0.50		1.3	C9H8O4	179.035	1.2	78	Hydroxycinnamic acids	−4.26		5.0	+++
4.682	Methydihydrojasmonic acid	0.15		0.0	C13H20O3	225.1135	1.6	43	Jasmonic acids	−3.96		7.4	+++
4.765	*p-*Coumaric acid	0.64		0.2	C9H8O3	163.0400	1.5	58	Hydroxycinnamic acids	−4.01		7.9	+++
5.672	Asiatic acid	0.09		3.0	C30H48O5	487.3429	5.7	98	Triterpenoids	−4.19		10.2	+++
5.7	Syringic acid	0.01		1.1	C9H10O5	197.0479	1.0	76	Hydroxybenzoic acids	N.D			-
6.226	Rosmarinic acid	0.03		1.0	C18H16O8	359.0772	2.4	145	Coumaric acids	−5.99		2.6	+
7.339	Eriodictyol	0.01		2.1	C15H12O6	287.0561	2.0	107	Flavanones	N.D			
7.627	Luteolin	0.21		4.5	C15H10O6	285.0404	1.4	107	Flavanones	−4.42		4.8	+++
7.809	protocatechuic acid-glucoside	0.43		8.5	C13H16O9	315.0722	−1.8	157	Hydrolyzable tannins	−4.35		5.4	+++
7.815	Isorhamnetin	0.46		0.3	C16H12O7	315.051	1.9	116	Flavonols	−4.31		3.6	+++
7.822	Trihydroxycinnamic acid derivative	0.41		0.2	C9H8O5	207.0636	0.8	98	Hydroxycinnamic acids	−3.63		4.2	+++
8.22	Umbelliferone	0.01		3.0	C9H6O3	161.0244	1.6	47	7-hydroxycoumarins	−4.42		3.7	+++
8.223	Esculin	0.01		0.1	C15H16O9	339.0722	−0.6	146	Coumarin glycosides	−4.30		14.0	+++
8.243	Apigenin	1.54		0.6	C15H10O5	269.0455	1.7	87	Flavones	−4.58		21.7	++
8.312	9,12,13- Trihydroxyoctadecadienoic acid	0.27		1.4	C18H32O5	327.2177	2.6	98	Lineolic acids	−3.73		5.6	+++
8.352	Hesperetin	0.08		7.4	C16H14O6	301.0718	2.4	96	O-methylated flavonoids	−4.77		8.6	++
8.415	Vanillic acid-O-hexoside	1.44		0.1	C14H18O9	329.0887	−1.5	146	Hydrolyzable tannins	−4.19		5.6	+++
8.422	12- Hydroxyjasmonic acid	0.05		2.2	C12H18O4	227.1277	0.4	75	Jasmonic acids	−4.06		21.3	+++
8.438	4-Methoxytectochrysin	0.03		0.9	C17H14O5	299.0914	3.3	65	Flavonols	−4.22		3.3	+++
8.494	Desdimethyl-octahydro-iso- cohumulone	0.15		0.7	C18H34O5	329.2333	3.8	71	monoterpenoids	−4.30		10.1	+++
8.653	Ladanein	0.02		4.2	C17H14O6	315.0863	2.0	85	O-methylated flavonoids	−3.85		2.3	+++
8.657	Diosmetin	1.96		1.0	C16H12O6	299.0557	1.7	96	O-methylated flavonoids	−4.50		10.1	++
8.703	Rosmanol	2.40		0.7	C20H26O5	345.1707	3.4	87	Diterpene lactones	−3.84		8.7	+++
8.753	Crisimaritin	0.18		0.3	C17H14O6	313.0718	2.0	85	O-methylated flavonoids	−4.36		8.5	+++
8.856	Artepillin C	1.41		2.1	C19H24O3	299.1653	5.4	58	Hydroxycinnamic acids	−4.42		5.1	+++
8.882	Isorhamnetin-3-O-glucoside	0.15		1.4	C31H28O15	641.1501	0.7	196	Flavonoid O-glycosides	N.D		0.5	-
8.958	Genkwanin	3.80		0.3	C16H12O5	283.0612	2.1	76	O-methylated flavonoids	−5.02		4.4	++
8.751	Rosmariquinone	3.80		0.3	C19H22O2	283.1693	4.9	34	Diterpenoids	−5.00		5.5	++
9.376	Isoquercitrin	0.21		10.4	C21H20O12	463.0882	0.4	207	Flavonoid O-glycosides	N.D		2.1	-
9.395	Homoplantaginin	0.39		14.4	C22H22O11	461.1098	0.8	175	Flavonoid O-glycosides	−4.24		5.0	+++
9.584	Carnosol	18.71		1.4	C20H26O4	329.1758	4.4	67	Diterpene lactones	−4.30		4.7	+++
9.631	Galdosol	0.73		13.9	C20H24O5	343.1551	4	84	Diterpene lactones	−4.41		11.1	+++
9.737	Rosmaridiphenol	1.05		0.6	C20H28O3	315.1966	5.9	58	Diterpenoids	−5.55		12.9	+
9.866	Carnosic acid	39.40		0.2	C20H28O4	331.1915	4.9	78	Diterpenoids	−4.87		11.0	++
10.081	Methyl carnosate	13.14		0.4	C21H30O4	345.2071	5.2	67	Diterpenoids	−5.26		0.7	++
10.084	Sugiol	0.11		0.0	C20H28O2	301.2162	5.6	38	Diterpenoids	−5.34		6.5	++
10.187	Nepitrine	0.25		1.0	C22H22O12	477.1067	0.5	196	Flavonoid O-glycosides	−4.16		10.1	+++
10.313	20-Deoxocarnosol	1.52		2.8	C20H28O3	317.2111	4.6	50	Diterpenoids	N.D			-
10.449	Hinokione	0.20		2.7	C20H28O2	299.2017	4.9	37	Diterpenoids	N.D			-
10.489	Micromeric acid	0.13		11.2	C30H46O3	453.3374	6.5	58	Triterpenoids	N.D			-
10.614	Betulinic acid	1.04		5.6	C30H48O3	455.3422	8.2	56	Triterpenoids	−6.64		0.6	+
10.916	Notohamosine B	0.01		3.3	C29H46O4	457.3315	5.6		Hydroxysteroids	−4.62		11.8	++
11.356	Anemosapogenin	0.16		8.0	C30H48O4	471.3480	7.5	78	Triterpenoids	−5.26		3.0	++

*PAMPA-BBB potential penetrability based on Könczöl et al. (69). –, not detected in acceptor; +, log Pe > −6.5; ++, log Pe > −5.5; +++, log Pe > −4.5 (60). Colors indicate the relative abundance of the identified compounds: green for highly abundant, yellow for middle abundant and red for low abundant*.

### UHPLC-Q-TOF-MS/MS Profiling Analysis of Plant Extracts

The RSFE extract was mainly composed of two major bioactive diterpenoids: carnosic acid and carnosol (see Table 1). Both compounds were previously identified in research works conducted by our group (31).

On the other hand, phenolic acids like *p-*coumaric acid, vanillic acid, and syringic acid are major compounds in the KD extract. These phenolic compounds were also reported in previous works by Bogucka-Kocka et al. (32) (see Table 2). The KD extract also contains a characteristic group of C_24_-steroids (bufanolides), such as bersaldegenin and bryophyllin A, among others, as reported for other KD extracts (33).

**Table 2:** Phytochemical profile, abundance (%), and BBB permeability of KD (Kalanchoe daigremontiana, kalanchoe UAE extract).

**Ret. Time**		**Abundance**	**RSD**	**Molecular**	**[M-H]- (m/z)**			**Family**	**PAMPA-BBB log**	**RSD**	**Cross BBB**
**(min)**	**Compound**	**(%)**	**(%)**	**formula**	**(theoretical)**	**Log P**	**TPSA**	**compound**	**Pe (cm s−1)**	**(%)**	**potential**
2.244	Protocatechuic acid	0.73	5.8	C7H6O4	153.0193	1.1	78	Hydroxybenzoic acids	−3.98	2.1	+++
4.741	*p-*Coumaric acid	25.85	5.4	C9H8O3	163.0401	1.5	58	Hydroxycinnamic acids	−4.15	4.6	+++
3.717	Vanillic acid	20.40	13.7	C8H8O4	167.0350	1.4	67	Methoxybenzoic acids	−4.26	10.7	+++
1.71	Gallic acid	0.40	0.5	C7H6O5	169.0142	0.7	98	Hydroxybenzoic acids	−4.46	11.0	+++
3.893	Caffeic acid	0.12	4.5	C9H8O4	179.0350	1.2	78	Hydroxycinnamic acids	−4.56	8.3	++
4.323	Ferulic acid	0.18	7.1	C10H10O4	193.0506	1.5	67	Hydroxycinnamic acids	−4.68	9.1	++
4.85	Syringic acid	12.72	9.3	C9H10O5	197.0455	1.0	76	Hydroxybenzoic acids	−5.42	2.3	++
0.56	Sinapic acid	0.59	3.3	C11H12O5	223.0612	1.5	76	Hydroxybenzoic acids	N.D		-
9.186	Kaempferol	0.78	7.1	C15H10O6	285.0405	1.9	107	Flavonols	−4.83	1.2	++
2.429	Quercetin	9.47	3.0	C15H10O7	301.0354	1.5	127	Flavonols	−7.09	0.1	+
6.446	Eupafolin	0.23	3.4	C16H12O7	315.0510	1.4	116	O-methylated flavonoids	−3.47	9.2	+++
2.432	Myricetin	0.09	1.2	C21H20O12	317.0303	1.2	148	Flavonols	N.D		-
4.32	Patuletin	1.27	0.3	C16H12O8	331.0459	2.1	137	Flavonols	N.D		-
8.487	Resibufagin	0.32	4.4	C24H30O5	397.2020	2.4	76	Bufanolides	N.D		-
5.246	Afzelin	0.04	8.6	C28H32O14	431.0984	1.2	166	Flavonoid O-glycosides	N.D		-
4.548	Bersaldegenin	0.77	5.4	C24H32O7	431.2075	0.2	124	Bufanolides	N.D		-
4.737	11α,19-dihydroxytelocinobufagin	0.20	2.1	C24H34O7	433.2232	3.63		Bufanolides	N.D		-
8.56	Kaempferol 3-O-β-D-glucoside	0.13	1.4	C21H20O11	447.0933	2.37	186	Flavonoid O-glycosides	N.D		-
7.119	Bersaldegenin-1,3,5-orthoacetate	0.21	0.3	C26H32O7	455.2075	4.87		Bufanolides	N.D		-
5.476	Hellebrigenol-3-monoacetate	0.81	1.7	C26H36O7	459.2388	1.6	113	Bufanolides	−4.70	5.5	++
8.083	3-O-β-D-glucopyranoside	5.40	2.7	C21H20O12	463.0882	0.4		Flavonoid O-glycosides	N.D		-
6.115	Bryophyllin A	14.18	5.8	C26H32O8	471.2024	0.6	112	Bufanolides	N.D		-
6.116	Bersaldegenin-3-acetate	0.54	3.2	C26H34O8	473.2181	1.3	130	Bufanolides	N.D		-
5.457	Kalandaigremoside B	1.68	3.4	C26H36O8	475.2337	7.2		Bufanolides	N.D		-
6.844	Daigremontianin	0.99	4.7	C26H30O9	485.1817	−0.3	129	Bufanolides	−4.53	2.2	++
6.791	Bryophyllin B	0.62	3.0	C26H32O9	487.1974	0.6	143	Bufanolides	N.D		-
6.854	Kalandaigremoside F	0.07	6.4	C26H34O9	489.2130	7.2		Bufanolides	N.D		-
5.897	Kalanchoside A	0.27	10.5	C30H42O10	561.2705	−0.1	163	Bufanolides	N.D		-
5.476	Kalandaigremoside C	0.38	3.0	C30H44O11	579.2811	7.2		Bufanolides	N.D		-
4.416	Paniculatin	0.42	7.2	C27H30O15	593.1512	−2.3	267	Isoflavonoid C-glycosides	N.D		-
5.676	Kalantuboside A	0.12	8.1	C32H42O12	617.2604	7.5		Bufanolides	N:D		-

*PAMPA-BBB potential penetrability based on Könczöl et al. (69). –, not detected in acceptor; +, log Pe > −6.5; ++, log Pe > −5.5; +++, log Pe > −4.5 (60). Colors indicate the relative abundance of the identified compounds: green for highly abundant, yellow for middle abundant and red for low abundant*.

Cooperage wood extracts (ASFE and LPLE) exhibit a similar phytochemical profile. ASFE extract was mainly composed of protocatechuic aldehyde, protocatechuic acid, and the flavanonol aromadendrin (see Table 3), whereas gallic acid and ellagic acid are major compounds in the LPLE extract (see Table 4). Various authors reported similar qualitative phenolic content in the raw material of other cooperage woods like chestnut, oak, and cherry (34–36).

**Table 3:** Phytochemical profile, abundance (%), and BBB permeability of ASFE (Robinia pseudoacacia, acacia SFE extract).

**Ret. Time**		**Abundance**	**RSD**	**Molecular**	**[M-H]- (m/z)**			**Family**	**PAMPA-BBB log**	**RSD**	**Cross BBB**
**(min)**	**Compound**	**(%)**	**(%)**	**formula**	**(theoretical)**	**Log P**	**TPSA**	**compound**	**Pe (cm s−1)**	**(%)**	**potential**
1.715	Pyrogallol	0.36	6.6	C6H6O3	125.0253	0.5	61	Pyrogallols	N.D		-
3.043	Protocatechuic aldehyde	13.01	4.4	C7H6O3	137.0254	1.1	58	Hydroxybenzoic acids	−5.75	1.1	+
5.012	Vanillin	0.39	5.4	C8H8O3	151.0408	1.2	47	Methoxyphenols	−4.15	8.9	+++
3.209	Protocatechuic acid	18.89	3.0	C7H6O4	153.0217	1.1	78	Hydroxybenzoic acids	−3.92	5.8	+++
2.666	Vanillic acid	1.68	5.3	C8H8O4	167.0366	1.4	67	Methoxybenzoic acids	−5.67	1.2	+
1.715	Gallic acid	3.92	2.8	C7H6O5	169.0155	0.7	98	Methoxybenzoic acids	−4.01	12.5	+++
5.903	Coniferaldehyde	0.12	7.8	C10H10O3	177.0575	1.5	47	Methoxyphenols	−4.24	1.7	+++
3.325	Caffeic acid	0.24	8.6	C9H8O4	179.0365	1.5	78	Hydroxycinnamic acids	−4.00	6.9	+++
8.325	Syringaldehyde	0.21	4.6	C9H10O4	181.0522	0.1	56	Methoxyphenols	−4.07	3.4	+++
5.351	Scopoletin	0.05	0.0	C10H8O4	191.0369	1.5	56	Hydroxycoumarins	−4.12	1.6	+++
3.335	Ferulic acid	0.07	5.8	C10H10O4	193.0526	1.5	67	Hydroxycinnamic acids	N.D		-
4.721	Syringic acid	0.95	2.7	C9H10O5	197.0479	1.0	76	Methoxybenzoic acids	−5.83	3.2	+
4.342	Sinapaldehyde	0.12	0.5	C11H12O4	207.0683	1.4	56	Methoxyphenols	N.D		-
3.849	Sinapic acid	0.04	5.8	C11H12O5	223.0631	1.5	76	Hydroxycinnamic acids	N.D		-
6.718	Trans-resveratrol	0.09	1.3	C14H12O3	227.0743	2.5	61	Stilbenes	N.D		-
8.037	Butein	8.27	9.7	C15H12O5	271.0640	2.8	98	Chalcones	−5.66	4.7	+
6.215	Hexadecanedioic acid	10.94	1.8	C16H30O4	285.0421	5.4	75	Long-chain fatty acids	N.D		-
9.402	Kaempferol	0.93	0.5	C15H10O6	285.2098	1.9	107	Flavonols	−5.64	3.0	+
6.96	Aromadendrin	13.48	0.2	C15H12O6	287.0586	1.8	107	Flavanonols	N.D		-
3.441	Catechin	1.78	7.5	C15H14O6	289.0755	0.4	110	Catechins	N.D		-
4.322	Coutaric acid	10.02	5.3	C13H12O8	295.0486	0.4	141	Hydroxycinnamic acids	N.D		-
5.341	Ellagic acid	0.19	3.4	C14H6O8	301.0016	1.1	134	Hydrolyzable tannins	N.D		-
3.335	Quercetin	1.08	1.6	C15H10O7	301.0380	1.5	127	Flavonols	−7.50	6.9	+
5.496	Hesperetin	6.75	2.6	C16H14O6	301.0748	2.4	96	O-methylated flavonoids	−5.22	2.2	++
2.802	Caftaric acid	0.11	9.8	C13H12O9	311.0442	0.1	162	Hydroxycinnamic acids	N.D		-
3.461	Fertaric acid	0.32	5.3	C14H14O9	325.0561	0.4	151	Hydroxycinnamic acids			-
3.615	Piceid			C20H22O8	389.1269	1.7	140	Stilbenes			
4.681	(-)-Epicatechin gallate	0.05	5.6	C22H18O10	441.0862	1.5	177	Catechins	N.D		-
11.381	ε-Viniferin	0.05	8.2	C28H22O6	453.1374	5.4	110	Stilbenes	N.D		-
4.351	Sanguisorbic acid dilactone			C21H10O13	469.006	−0.4	221	Hydroxybenzoic acids			
10.461	Triterpene*	0.04	5.5	C29 H44 O5	471.3124	3.6	84	Triterpenoids	−4.97	11.8	++
9.821	Corosolic acid	0.08	1.5	C30 H48 O4	471.3461	6.4	78	Triterpenoids	−5.22	4.3	++
4.564	Bis-O-galloyl-glucose	0.07	0.0	C20H20O14	483.081	−0.8	177	Tannins	N.D		
10.917	Lucyin A			C30 H46 O5	485.3275	5.3	95	Triterpenoids			
10.799	Cucurbitacin S			C30 H42 O6	497.2917	3.2	101	Oxosteroids			
10.923	Ganolucidic acid B	0.12	0.4	C30 H46 O6	501.3249	3.8	112	Triterpenoids	N.D		-
8.849	Ganolucidic acid C	0.07	2.3	C30 H46 O7	517.3183	3.1	132	Triterpenoids	N.D		-
5.042	Procyanidin B2	5.47	2.2	C30H26O12	577.1373	2.4	221	Biflavonoids	N.D		-
8.345	Hesperidin	0.02	1.0	C28H34O15	609.186	−1.1	234	Flavonoid O-glycosides	N.D		-
6.457	Kaempferol diacetylglucoside rhamnoside	0.04	7.1	C31H34O17	677.1735	0.1	245	Flavonoid O-glycosides	N.D		-

* (23S,24S)-17,23-Epoxy-24,29-dihydroxy-27-norlanost-8-ene-3,15-dione.

*PAMPA-BBB potential penetrability based on Könczöl et al., (69). –, not detected in acceptor; +, log Pe > −6.5; ++, log Pe > −5.5; +++, log Pe > −4.5 (60). Colors indicate the relative abundance of the identified compounds: green for highly abundant, yellow for middle abundant and red for low abundant*.

**Table 4:** Phytochemical profile, abundance (%), and BBB permeability of LPLE (Nothofagus pumilio, lenga PLE extract).

**Ret. Time**		**Abundance**		**RSD**	**Molecular**	**[M-H]- (m/z)**			**Family**	**PAMPA-BBB log**	**RSD**	**Cross BBB**
**(min)**	**Compound**	**(%)**		**(%)**	**formula**	**(theoretical)**	**Log P**	**TPSA**	**compound**	**Pe (cm s−1)**	**(%)**	**potential**
1.715	Pyrogallol	2.90		9.6	C6H6O3	125.0253	0.5	61	Pyrogallols	N.D		-
3.043	Protocatechuic aldehyde	0.18		13.7	C7H6O3	137.0254	1.1	58	Hydroxybenzoic acids	−5.13	8.6	++
5.012	Vanillin	0.22		2.3	C8H8O3	151.0408	1.2	47	Methoxyphenols	−4.48	0.3	+++
3.209	Protocatechuic acid	0.21		9.9	C7H6O4	153.0217	1.1	78	Hydroxybenzoic acids	−3.86	4.0	+++
2.666	Vanillic acid	0.07		7.6	C8H8O4	167.0366	1.4	67	Methoxybenzoic acids	−5.87	7.4	+
1.715	Gallic acid	31.67		8.2	C7H6O5	169.0155	0.7	98	Methoxybenzoic acids	−4.16	5.2	+++
5.903	Coniferaldehyde	0.51		13.7	C10H10O3	177.0575	1.5	47	Methoxyphenols	−4.65	4.2	++
3.325	Caffeic acid	0.04		1.5	C9H8O4	179.0365	1.5	78	Hydroxycinnamic acids	N.D		-
8.325	Syringaldehyde	0.02		2.5	C9H10O4	181.0522	0.1	56	Methoxyphenols	−4.87	9.7	++
5.351	Scopoletin	0.09		8.4	C10H8O4	191.0369	1.5	56	Hydroxycoumarins	−4.55	1.2	++
3.335	Ferulic acid				C10H10O4	193.0526	1.5	67	Hydroxycinnamic acids			
4.721	Syringic acid	8.62		7.3	C9H10O5	197.0479	1.0	76	Methoxybenzoic acids	−6.84	1.5	+
4.342	Sinapaldehyde				C11H12O4	207.0683	1.4	56	Methoxyphenols			
3.849	Sinapic acid				C11H12O5	223.0631	1.5	76	Hydroxycinnamic acids			
6.718	Trans-resveratrol				C14H12O3	227.0743	2.5	61	Stilbenes			
8.037	Butein	1.19		1.0	C15H12O5	271.0640	2.8	98	Chalcones	−4.78	3.1	++
6.215	Hexadecanedioic acid	0.99		4.7	C16H30O4	285.0421	5.4	75	Long-chain fatty acids	N.D		-
9.402	Kaempferol	8.76		1.0	C15H10O6	285.2098	1.9	107	Flavonols	−4.67	9.8	++
6.96	Aromadendrin	2.58		2.5	C15H12O6	287.0586	1.8	107	Flavanonols	N.D		-
3.441	Catechin	0.05		7.4	C15H14O6	289.0755	0.4	110	Catechins	N.D		-
4.322	Coutaric acid	0.07		18.9	C13H12O8	295.0486	0.4	141	Hydroxycinnamic acids	N.D		-
5.341	Ellagic acid	23.83		3.8	C14H6O8	301.0016	1.1	134	Hydrolyzable tannins	N.D		-
3.335	Quercetin	0.05		0.9	C15H10O7	301.0380	1.5	127	Flavonols	−7.61	1.22	+
5.496	Hesperetin	1.50		10.2	C16H14O6	301.0748	2.4	96	O-methylated flavonoids	−5.30	8.9	++
2.802	Caftaric acid	0.78		1.1	C13H12O9	311.0442	0.1	162	Hydroxycinnamic acids	N.D		-
3.461	Fertaric acid	0.15		0.1	C14H14O9	325.0561	0.4	151	Hydroxycinnamic acids	N.D		-
3.615	Piceid	0.22		0.5	C20H22O8	389.1269	1.7	140	Stilbenes	N.D		-
4.681	(-)-Epicatechin gallate	0.04		1.1	C22H18O10	441.0862	1.5	177	Catechins	N.D		-
11.381	ε-Viniferin	0.09		3.1	C28H22O6	453.1374	5.4	110	Stilbenes	N.D		-
4.351	Sanguisorbic acid dilactone	4.50		6.2	C21H10O13	469.006	−0.4	221	Hydroxybenzoic acids	N.D		-
10.461	Triterpene*	2.69		5.7	C29 H44 O5	471.3124	3.6	84	Triterpenoids	−5.05	7.4	++
9.821	Corosolic acid	2.69		6.3	C30 H48 O4	471.3461	6.4	78	Triterpenoids	−5.07	6.7	++
4.564	Bis-O-galloyl-glucose	3.04		4.1	C20H20O14	483.081	−0.8	177	Tannins	N.D		-
10.917	Lucyin A	0.12		9.0	C30 H46 O5	485.3275	5.3	95	Triterpenoids	−4.74	4.8	++
10.799	Cucurbitacin S	0.10		11.7	C30 H42 O6	497.2917	3.2	101	Oxosteroids	N.D		-
10.923	Ganolucidic acid B	1.63		1.7	C30 H46 O6	501.3249	3.8	112	Triterpenoids	N.D		-
8.849	Ganolucidic acid C	0.01		6.6	C30 H46 O7	517.3183	3.1	132	Triterpenoids	N.D		-
5.042	Procyanidin B2	0.30		4.6	C30H26O12	577.1373	2.4	221	Biflavonoids	N.D		-
8.345	Hesperidin	0.10		4.2	C28H34O15	609.186	−1.1	234	Flavonoid O-glycosides	N.D		-
6.457	Kaempferol diacetylglucoside rhamnoside	0.00		7.2	C31H34O17	677.1735	0.1	245	Flavonoid O-glycosides	N.D		-

* (23S,24S)-17,23-Epoxy-24,29-dihydroxy-27-norlanost-8-ene-3,15-dione.

*PAMPA-BBB potential penetrability based on Könczöl et al. (69). –, not detected in acceptor; +, log Pe > −6.5; ++, log Pe > −5.5; +++, log Pe > −4.5 (60). Colors indicate the relative abundance of the identified compounds: green for highly abundant, yellow for middle abundant and red for low abundant*.

With regard to T33 extract, the hydroxycinnamic acid ethyl caffeate was the main compound, followed by other phenolic acids such as gallic acid and *p-*coumaric acid (see Table 5), as reported by Suárez Montenegro et al. (18).

**Table 5:** Phytochemical profile, abundance (%), and BBB permeability of T33 (Cyphomandra betacea, tamarillo PLE extract).

**Ret. Time**		**Abundance**	**RSD**	**Molecular**	**[M-H]- (m/z)**			**Family**	**PAMPA-BBB log**	**RSD**	**Cross BBB**
**(min)**	**Compound**	**(%)**	**(%)**	**formula**	**(theoretical)**	**Log P**	**TPSA**	**compound**	**Pe (cm s−1)**	**(%)**	**potential**
3.043	p-salycilic acid	4.83	7.3	C7H6O3	137.0244	3.7	58	Hydroxybenzoics acid	−4.75	5.9	++
3.381	*p-*Coumaric acid	5.99	2.4	C9H8O3	163.0401	1.5	58	Hydroxycinnamic acids	−5.05	0.2	++
2.993	Gallic acid	6.41	1.1	C7H6O5	169.0142	0.7	98	Hydroxybenzoic acids	−4.63	2.1	++
4.14	Caffeic acid	3.35	2.0	C9H8O4	179.0350	1.2	78	Hydroxycinnamic acids	−4.07	5.0	+++
6.224	Syringaldehyde	1.63	10.1	C9H10O4	181.0506	1.1	56	Methoxyphenols	−4.55	1.1	++
0.611	Quinic acid	7.86	3.2	C7H12O6	191.0561	−2.4	118	Cyclic alcohols	−5.44	9.7	++
7.676	Ethyl caffeate	35.97	2.7	C11H12O4	207.0663	2.6	67	Hydroxycinnamic acids	−4.73	11.1	++
5.621	Sinapic acid	1.57	0.9	C11H12O5	223.0612	1.5	76	Hydroxybenzoic acids	N.D		-
3.557	O-acetyl-quinic acid	8.91	5.5	C9H14O7	233.0667	−2.4	124	Cyclic alcohols	N.D		-
11.38	Phytuberin	1.07	3.7	C17H26O4	293.1758	2.3	45	Sesquiterpenoids	−4.51	0.9	++
4.71	Caffeoylshikimic acid	4.27	2.9	C16H16O8	335.0772	0.9	147	Hydroxycinnamic acids	−5.46	6.0	++
2.983	Caffeoyl hexoside	0.52	4.5	C15H18O9	341.0878	−0.6	157	Hydroxycinnamic acid	N.D		-
3.49	Caffeoylquinic acid (chlorogenic acid)	4.42	5.3	C16H18O9	353.0878	−0.4	165	Cyclic alcohols	−4.99	7.5	++
6.125	Rosmarinic acid	2.23	2.2	C18H16O8	359.0772	2.4	145	Coumaric acids	−5.16	4.8	++
2.112	Syringic acid hexoside	2.73	8.4	C15H20O10	359.0984	−1.0	155	Hydrolyzable tannins	N.D		-
5.098	Methyl caffeoyl quinate	1.50	11.4	C17H20O9	367.1034	1.9	200	Cyclic alcohols	−5.20	2.2	++
6.098	Hexosyl methyl ferulate	0.44	8.9	C17H22O9	369.1191			Hydroxycinnamic acids	N.D		-
5.853	Methyl feruloyl quinate	5.44	6.1	C18H22O9	381.1191	0.2	143	Hydroxycinnamic acids	N.D		-
5.555	Isoquercitrin	0.58	9.7	C21H20O12	463.0877	0.4	207	Flavonoid O-glycosides	N.D		-
5.409	Rutin	0.23	0.4	C27H30O16	609.1461	−1.3	266	Flavonoid O-glycosides	N.D		-

*PAMPA-BBB potential penetrability based on Könczöl et al. (69). –, not detected in acceptor; +, log Pe > −6.5; ++, log Pe > −5.5; +++, log Pe > −4.5 (60). Colors indicate the relative abundance of the identified compounds: green for highly abundant, yellow for middle abundant and red for low abundant*.

### *In vitro* Neuroprotective Activity Assays

#### Anticholinesterase *in vitro* Assay

The results for *in vitro* neuroprotective experiments were expressed as IC_50_ values (concentration of extract that causes 50% inhibition), which means that the extract with the lowest IC_50_ value is the one with the highest neuroprotection capacity. The results of enzymatic inhibition by AChE and BChE are shown in Table 6. As can be seen, ASFE extract exhibits strong cholinesterase inhibition capacity, with IC_50_ values comparable to galantamine (positive control). These results clearly improve the weak enzymatic inhibition activity of AChE and BchE (26.32 and 31.47 ID%, respectively) of *Robinia pseudoacacia* L. extract in chloroform: methanol (1:1, v/v) reported by Orhan et al. in 2004 (37), demonstrating the efficiency of SFE approaches that enhance the recovery of bioactive compounds (38). Satisfactory cholinesterase inhibitory potential values were obtained for the other tested extracts (RSFE, KD, T33, and LPLE), compared to other natural cholinesterase inhibitors proposed by several authors in the literature, which showed lower anticholinesterase enzymatic capacity (39, 40). Hassan et al. (41) reported antioxidant and AChE inhibition activity of methanolic and aqueous conventional extracts from tamarillo, which showed lower anticholinesterase capacity than our T33 extract. These differences may be attributed to higher total phenolic and flavonoid content in the T33 extract obtained by a PLE procedure (18).

**Table 6:** IC_50_ values from *in vitro* assays of different extracts using anticholinesterase (AChE and BChE), anti-inflammatory (LOX), and antioxidant (ABTS, ROS, and RNS) assays.

**Extract**	**AChE**		**BChE**		**LOX**		**ABTS**		**ROS-ORAC**		**RNS**
(IC50 μg mL^**−1**^)											
RSFE	107.85^a^±8.39		54.89^c^±1.53		9.82^d^±0.88		35.63^a^±1.14		4.51^a^±0.23		95.35^e^±6.45
KD	42.95^c^±2.29		8.26^d^±0.75		44.90^bc^±0.45		1.77^e^±0.20		1.12^e^±0.02		348.01^d^±25.66
T33	97.46^b^±6.82		85.46^a^±2.68		48.30^b^±1.66		6.33^c^±0.01		2.54^b^±0.14		599.00^c^±5.92
LPLE	49.57^c^±1.09		72.84^b^±4.91		39.14^c^±2.72		4.22^d^±0.37		2.65^b^±0.08		ND ±
ASFE	4.23^d^±0.11		1.20^e^±0.06		4.37^d^±0.29		0.11^f^±0.01		1.56^c^±0.11		3218.24^a^±358.62
**Positive control**											
Galantamine	0.40^d^±0.00		2.41^e^±0.00
Quercetin					125.73^a^±10.71
Ascorbic acid							25.02^b^±0.34		1.34^d^±0.11		1100^b^±14.0

*Different letters in the same column show significant differences (p <0.05). RSFE (Rosmarinus officinalis L., SFE extract); KD (Kalanchoe daigremontiana, kalanchoe UAE extract); T33 (Cyphomandra betacea, tamarillo PLE extract); LPLE (Nothofagus pumilio, lenga PLE extract); ASFE (Robinia pseudoacacia, acacia SFE extract)*.

Several flavonoids and phenolic acids identified in the cooperage wood extracts (LPLE and ASFE) are reported as cholinesterase enzymatic inhibitors (42, 43). Thus, two major compounds in ASFE samples, aromadendrin and protocatechuic acid, are reported as selective AChE and BChE inhibitors, respectively (44, 45), which might explain the significantly higher anticholinergic activity of ASFE when compared to LPLE extracts.

The anticholinesterase capacity of KD and T33 extract may be attributed to the high abundance of phenolic acids such as *p-*coumaric acid and ethyl caffeate, respectively. Moreover, KD extract presents high amounts of flavonols like quercetin with larger dual AChE and BChE inhibition capacity (46). Preliminary results obtained by our group showed higher *in vitro* neuroprotective potential from KD extracts enriched in the phenolic compound, compared to the KD extracts enriched with bufadienolides (20). For this reason, the phenolic fraction of KD extract was considered for neuroprotective evaluation in this study. The amount and position of hydroxyl groups at the phenyl ring in the phenolic compound structure seem to be related to cholinesterase inhibition potential. Hydroxyl groups promote hydrogen bond formation with specific amino acids in the active sites of enzymes. Interestingly, an increasing number of hydroxyl groups on the side phenyl rings would result in more AChE inhibition and lower BChE inhibition (kaempferol and myricetin) (47). This fact could explain the differences between the AChE and BChE inhibition potential of each tested extract. Moreover, the presence of sugar moieties represents constraints for the accommodation of the glycosylated phenolic compound and enzyme active site, resulting in lower inhibition capacity (47).

#### Anti-Inflammatory *in vitro* Assay

Regarding LOX inhibition, all the studied extracts significantly improved the inhibition capacity of quercetin positive control, presenting IC_50_ values close to 50 μg ml^−1^ and below. RSFE and ASFE extracts exhibited the highest anti-inflammatory capacity (see Table 6). Lončarić et al. have recently reviewed works on plant families with potential LOX inhibitory activity (48), reporting a broad range of LOX inhibition values, ranging from 0.01 to >500 μg ml^−1^ IC_50_. In agreement with our results, extracts like ASFE and RSFE from *Fabaceae* and *Lamiaceae* families, respectively, are reported as major LOX inhibitors. Therefore, LOX inhibition assays carried out in this work yielded promising results, particularly for ASFE extract. Several works highlight phenolic compounds as natural compounds with elevated anti-inflammatory properties, including LOX inhibition capacity (43, 49, 50). Sadik et al. proposed some molecular features of flavonoids that influence LOX inhibition and cholinesterase inhibition capacity, such as the presence of hydroxyl groups. Hydroxylated flavonoids like quercetin or luteolin improve inhibition capacity by interposition between the hydrophobic cavity at the enzyme active site. Nevertheless, highly hydroxylated flavonoids like myricetin lower the hydrophobicity of the molecule and reduce the inhibitory mechanism of the phenolic compounds. Moreover, the presence of catechol moiety and/or the 2,3-double bond in the C ring of phenolic compounds rise the planarity of the molecule and also improves inhibition capacity (quercetin). Instead, the presence of sugar moiety also depresses the inhibitory potential of glycosylated flavonoids. LOX possesses ferric iron at the active site, thereupon another inhibitory mechanism by which flavonoids can act is their iron-chelating capacity (49, 51). The high LOX inhibition potential of ASFE extract may be attributed to its levels of lutein, a chalcone that is considered as a flavonoid biosynthetic precursor with an open structure and anti-inflammatory potential (52).

In the present work, enzymatic inhibition by LOX was measured to evaluate the protective effect against neuroinflammation; however, the *in vivo* relationship between LOX enzyme and pro-inflammatory cytokines must be taken into account, since LOX activity products could stimulate pro-inflammatory cytokine generation (IL-6, TNF-α, and IL-1β among others) (53). Thus, anti-inflammatory cell-based assays focused on pro-inflammatory cytokine observation frequently use macrophages (e.g., Raw 264.7 and THP-1 cell lines) that constitutively express some LOX isoforms (54, 55). Thus, some anti-inflammatory results could arise from LOX inhibition. In fact, LOX inhibitors have shown the capacity to reduce the production of some inflammatory cytokines in the cells of the central nervous system model (53). Some studies showed anti-inflammatory properties of *Robinia pseudoacacia* (56) and *Cyphomandra betacea* (57) in Raw 264.7 cell lines, whereas *Kalanchoe* species showed anti-inflammatory capacity in animal models also (58).

#### Antioxidant *in vitro* Assay

Two widely used antioxidant activity assays, namely ABTS and ORAC, were applied to test the target extracts. As can be seen in Table 6, the ASFE extract showed the highest antioxidant capacity in the ABTS assay. All the tested extracts against ABTS radical showed improved antioxidant capacity compared to ascorbic acid, except for RSFE extract. With regard to the ORAC probe, the major ROS scavenging capacity was exhibited by the KD extract, with statistical differences compared to a positive control (ascorbic acid) (Table 6). On the other hand, RSFE followed by KD extract exhibited the highest RNS scavenging activity. All tested extracts in the RNS assay showed better RNS scavenging capacity than ascorbic acid, except for ASFE extract. The above-discussed results of antioxidant capacity are in line with the data reported in the literature for tamarillo (41), kalanchoe (32), and acacia (59) extracts. Several researchers suggested two mechanisms for the antioxidant potential of polyphenolic structures: (1) phenolic hydroxyl groups are capable of donating a hydrogen atom or an electron to a free radical (in fact, the number of hydroxyl groups directly acted as the maker of antioxidant and free radical scavenging activity *via* hydrogen donation to form stable compounds); and (2) their extended conjugated aromatic complex is prone to delocalize an unpaired electron from ROS/RNS species (60). Interestingly, the presence of sugar moiety also reduces the antioxidant potential (60).

The strong antioxidant properties found in ASFE and KD extracts may be attributed to their high content of phenolic acids. In particular, their high content of hydroxycinnamic and hydroxybenzoic acids, such as protocatechuic and syringic acid, is suggested to enhance their antioxidant capacity (61). The remarkable RNS activity observed for RSFE extract is most probably due to the presence of terpenoid compounds. Previous works from our group suggest that terpenoids exhibit stronger RNS scavenging capacity than phenolic compounds (24).

### BBB Permeability Evaluation

**Figure 1:**
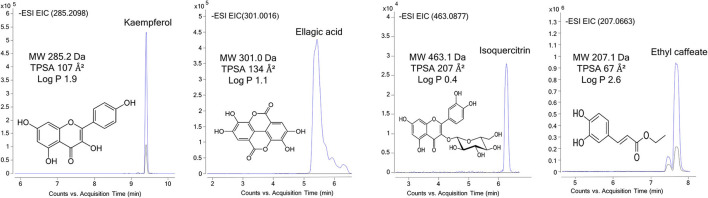
UHPLC-Q-TOF-MS/MS extracted ion chromatograms of representative bioactive compounds (kaempferol, isoquercitrin, ellagic acid, and ethyl caffeate) from T33 (Cyphomandra betacea, tamarillo PLE extract), ASFE (Robinia pseudoacacia, acacia SFE extract), and LPLE (Nothofagus pumilio, lenga PLE extract) extracts detected in the donor (blue) and acceptor plates (dark) of the PAMPA-BBB model.

The permeability study of bioactive molecules across the BBB is considered an essential approach to screen for neuroprotective compounds capable of reaching the central nervous system (CNS). Several molecular parameters, such as lipophilicity (calculated as oil/water partition coefficient, logP), topological polar surface area (TPSA), and molecular weight (MW), affect diffusion across BBB (Figure 1). Other molecular properties, such as hydrogen bond donors, octanol/water partition coefficient at a specific pH, and ionization of compounds (pKa), among others, should also be taken into account (62, 63). In the present work, PAMPA-BBB Log Pe was calculated for 113 different compounds from the studied plant matrices. Molecular properties, such as molecular weight, partition coefficient (LogP), and TPSA, were also considered to evaluate their influence on BBB permeability (Tables 1–5). Target phytochemicals were classified into chemical families according to their structural similarity.

**Figure 2:**
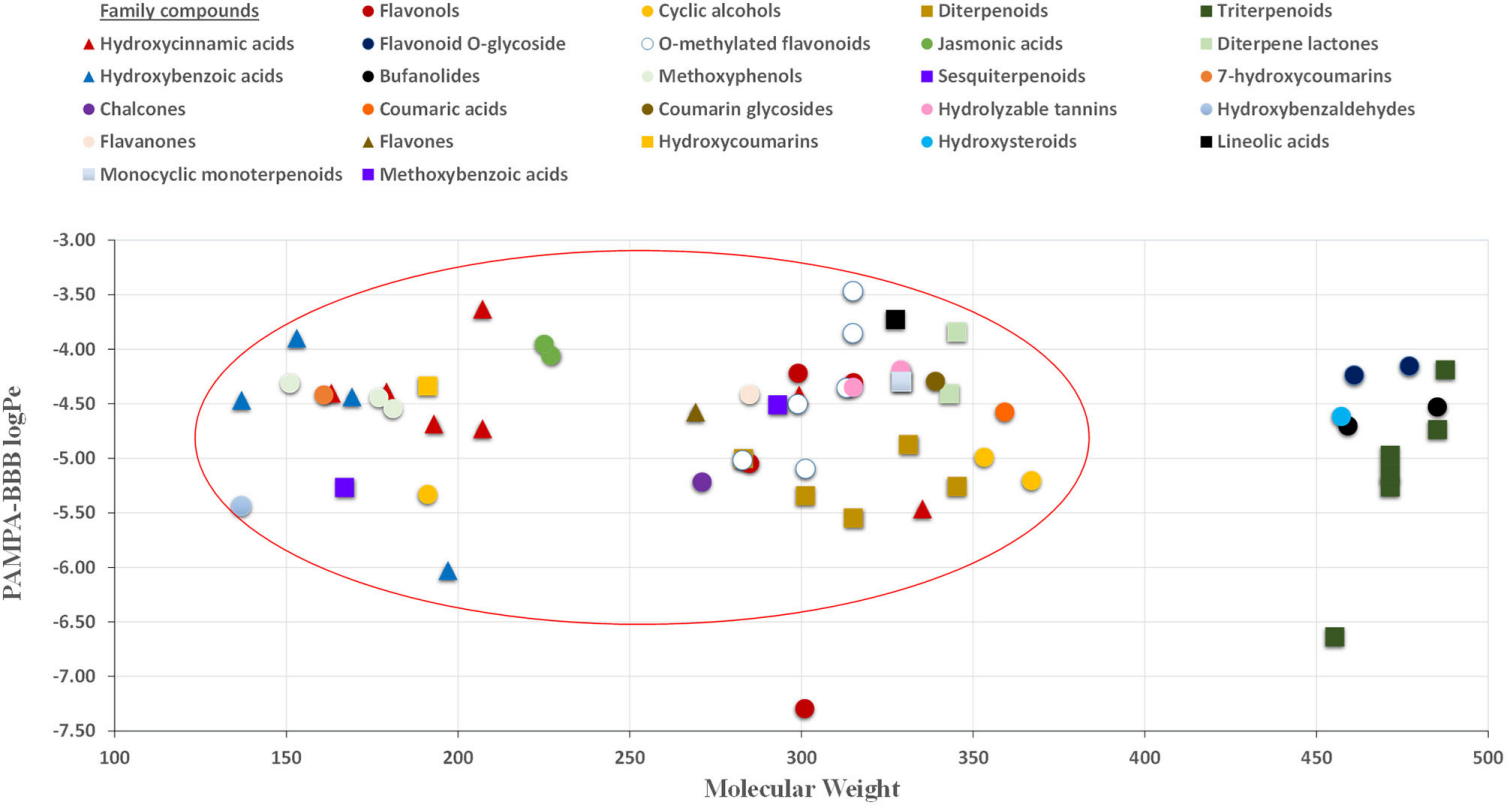
Relationship between PAMPA-BBB logPe and MW (molecular weight) values.

**Figure 3:**
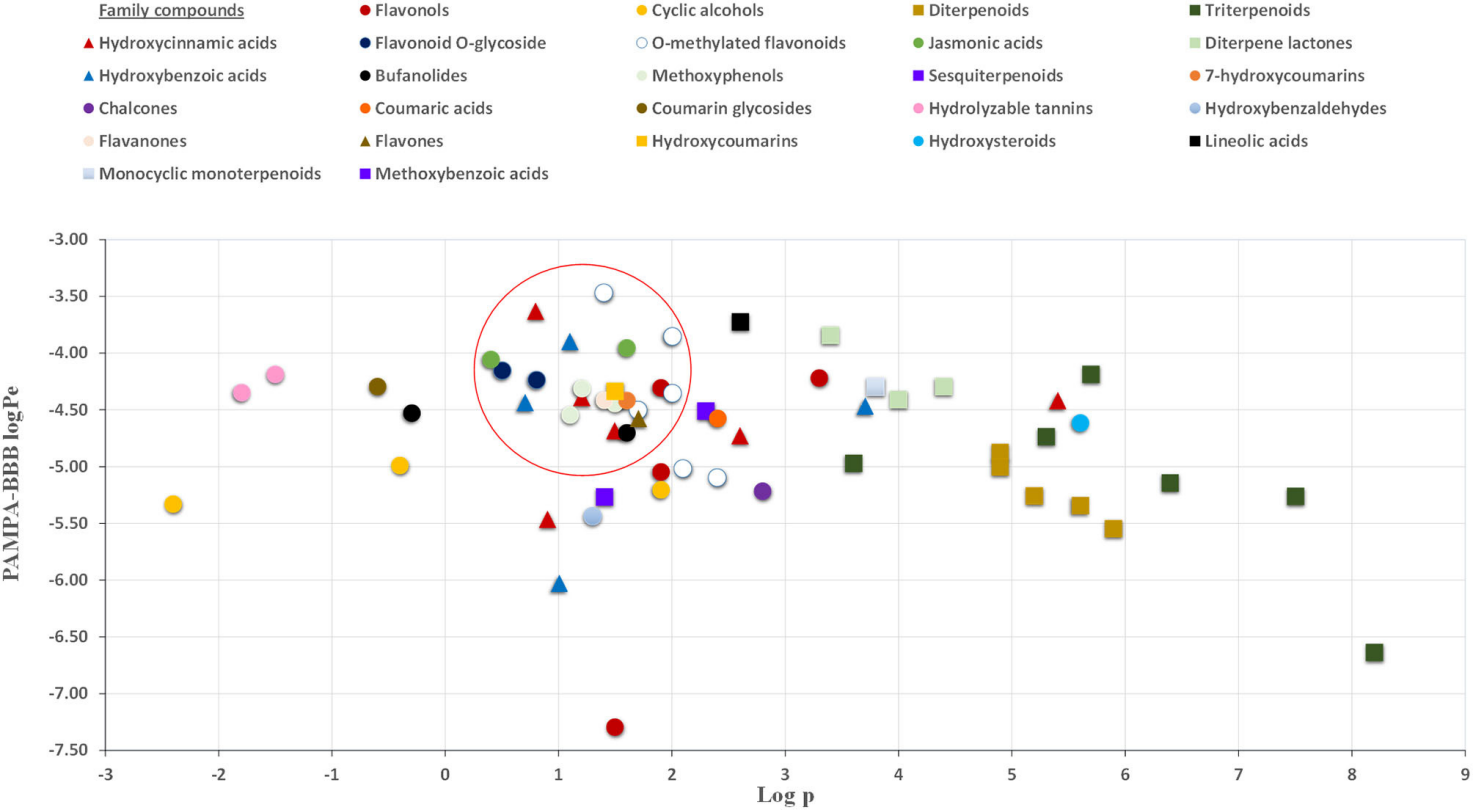
Relationship between PAMPA-BBB logPe and Log (octanol-water partition coefficient) values.

As can be seen in Figure 2, almost all small compounds with MW below 450–500 Da have similar possibilities to cross the BBB, in agreement with a previous study (64). In fact, studied compounds with MW above 500 Da, such as bufanolides and glycosylated flavonoids, were not detected in the acceptor plate. Regarding lipophilicity, some authors, for example, Agatonovic-Kustrin et al. (65), highlight that molecules with higher Log*P*-values penetrate better through the BBB due to the lipophilic nature of this physiological barrier. However, other authors consider that molecules with log*P*-values between 0 and 3 exhibit increased BBB accessibility (64, 66), in agreement with our result shown in Figure 3. Thus, compounds with the highest BBB diffusion, such as methylated flavonoids and phenolic acids, present log*P*-values between 0 and 2 (see Figure 3).

**Figure 4:**
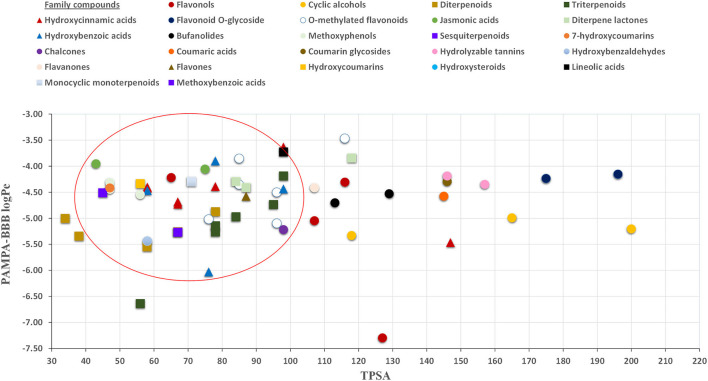
Relationship between PAMPA-BBB logPe and TPSA (topological polar surface area).

The effect of TPSA on the BBB perfusion capacity was previously studied by Hitchcock (64), suggesting 90 Å^2^ as the limit value for TPSA (64). In this regard, our results showed that most of the compounds capable of crossing the proposed *in vitro* BBB model exhibit TPSA values lower than 90 Å^2^ (see Figure 4). In fact, the identified molecules with large TPSA values, such as glycosylated flavonoids, were not capable of crossing the PAMPA-BBB. In this regard, in terms of neuroprotective value, the presence of glycosylated moieties in the molecular structure not only impairs their bioactive capacity, as discussed in Section 2.3, but also decreases their BBB perfusion due to their high MW and TPSA, and low log*P*-values (Figure 1).

The presence of low molecular weight phenolic compounds, such as protocatechuic acid and ferulic acid, among other phenolic acids, was reported in brain tissues after oral administration in *in vivo* experiments with rats (67). Moreover, charged molecules like phenolic acids could enhance BBB permeation by interactions with hydrogen bond donors through carboxylic acid groups (68). On the other hand, Könczöl et al. (69) studied more complex phenolic compounds from *Ginkgo biloba* L. extract in a PAMPA-BBB model. Similar PAMPA-BBB logPe permeability results were reported for apigenin (−5.35), kaempferol (−5.13), and isorhamnetin (−5.46), the compounds that are also present in our extracts. Glycosylated and <500 Da compounds were not detected in the acceptor plate. In the present study, quercetin and galantamine (current treatment of AD) were considered as positive controls for anti-inflammatory and anticholinesterase *in vitro* activity assays, respectively; however, the permeability of BBB to quercetin (LogPe = −7.02 ± 0.08) is limited compared to other bioactive compounds in the target extracts.

In summary, tentatively identified phytochemicals in the studied plant matrices were shown to have promising permeability across *in vitro* BBB, in comparison with other natural molecules and therapeutic drugs (e.g., logPe (cm s^−1^) galantamine =–5.35 ± 0.02) (70). It should be noted that some of the studied compounds like daigremontianin could not be found in the acceptor plate, as they do not follow the physicochemical BBB permeability rules mentioned earlier.

### Cell Culture *in vitro* Cytotoxicity

**Figure 5:**
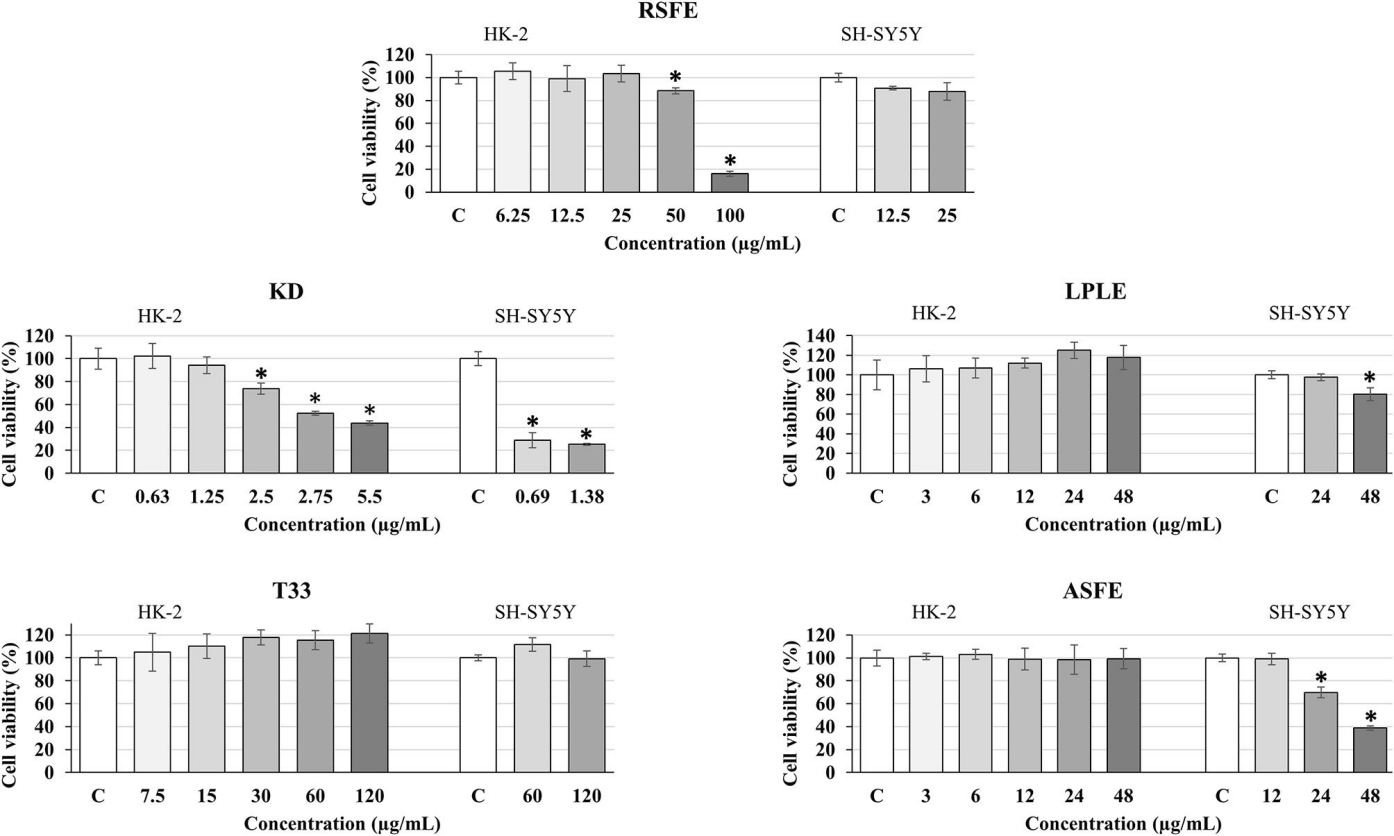
Effect of different concentrations of extracts: RSFE (*Rosmarinus officinalis* L., SFE extract), KD (*Kalanchoe daigremontiana*, kalanchoe UAE extract), LPLE (*Nothofagus pumilio*, lenga PLE extract), T33 (*Cyphomandra betacea*, tamarillo PLE extract), and ASFE (*Robinia pseudoacacia*, acacia SFE extract) on cell viability in HK-2 (human proximal tubular epithelial cells) and differentiated SH-SY5Y human neuroblastoma cells. Each bar is the mean of three determinations ± SD. * Denotes statistical differences when compared to the percentage of living cells in extract-treated cells with control (EtOH-treated cells which were considered as 100%). (*p < 0.05; ANOVA test, mean values from experimental results were compared by Tukey's HSD tests).

The *in vitro* cytotoxicity of the plant extracts was evaluated by the use of two different cell culture models: HK-2 and SH-SY5Y cell lines. These cell lines were selected because they are considered to be suitable and validated models to predict *in vitro* toxicity in common (HK-2) and neuronal-like (SH-SY5Y) human cells (28, 71). First, toxicity was determined in the HK-2 cell line. As can be observed in Figure 5, only two extracts showed some toxicity: RSFE (at 50 and 100 μg ml^−1^) and KD (at 2.5–5.5 μg ml^−1^). Based on these results, the maximum non-toxic concentrations for all extracts were selected to perform the cytotoxicity experiments in SH-SY5Y cells. As can be seen in Figure 5, T33 and RSFE extracts maintained maximum cell viability at the highest concentrations used (120 μg ml^−1^ for T33 and 25 μg ml^−1^ for RSFE). However, ASFE and LPLE extracts showed cytotoxicity at some of the tested concentrations (24 and 48 μg ml^−1^ for ASFE and 48 μg ml^−1^ for LPLE). Finally, KD extract showed high cytotoxicity at the lowest concentration tested (0.69 μg ml^−1^). These results identified T33 and RSFE as non-toxic extracts, ASFE and LPLE as mild-cytotoxic extracts, and KD extract as a highly toxic extract. Previous studies have reported cytotoxic effects of different phenolic extracts in SH-SY5Y cells. For example, Lantto et al. (72) reported toxic concentrations of extracts from basil (2,000 μg ml^−1^), juniper berry (10 μg ml^−1^), and laurel (10 μg ml^−1^), while parsley extracts were non-toxic up to 2,000 μg ml^−1^. Moreover, Sereia et al. (73) found toxic effects of *Poincianella pluviosa, Limonium brasiliense*, and *Stryphnodendron adstringens* extracts for SH-SY5Y cells at concentrations of 125, 500, and 1,000 μg ml^−1^, respectively, after 24 h of incubation (73). These authors did not find evidence for the relationship between phenolic compounds and toxicity in SH-SY5Y cells, and phenolic acids have been suggested as safe for SH-SY5Y cells (74). Other plant matrices, such as *Dunaliella salina* and *Pistacia lentiscus* L., have shown toxicity in SH-SY5Y cells when using concentrations above 50 μg ml^−1^ (after 48 h of incubation) and 56.4 μg ml^−1^ (after 24 h of incubation) (75, 76). Finally, the high cytotoxic results observed for KD extract are in line with Stefanowicz-Hajduk et al. (33), who demonstrated that bufadienolides obtained from *K. daigremontiana* are a potential tool against the proliferation of *in vitro* human cancer cell lines. In fact, the water and the ethanolic fraction of *K. daigremontiana* extract (which were mainly composed of phenolic compounds) did not show any toxic effect in cancer cell lines; on the other hand, the dichloromethane fraction enriched in bufadienolides presented the strongest activity against all the tested cell lines (33). Based on the chemical composition of the KD extract, the high concentration of bufadienolides may be responsible for the toxicity observed. For this reason, bufadienolides may be considered as compounds with large cytotoxic and neurotoxic effects (77).

## Conclusion

In this research, extracts obtained from various natural biomass samples by different green extraction approaches (SFE, PLE, and UAE) were evaluated through a set of *in vitro* bioactivity assays (AChE, BChE, LOX ABTS, ROS, and RNS) to investigate their neuroprotective potential. ASFE extract was demonstrated to have the greatest *in vitro* anticholinergic, anti-inflammatory, and antioxidant potential, being a valuable source of phenolic compounds. BBB permeability and cytotoxicity of the extracts have been explored to provide further evidence of their bioactive potential. In this regard, T33 and RSFE extracts can be considered as non-cytotoxic, as shown by cell viability assays in HK-2 and SH-SY5Y cell lines, whereas ASFE and LPLE extracts showed a slight cytotoxic effect. Despite the presence of highly valuable bioactives, KD extract exhibited high cytotoxicity.

Moreover, a broad range of evaluated phytochemicals identified in the target extracts showed *in vitro* capacity to reach the CNS by crossing the BBB and exert their neuroprotection activity. Some physicochemical properties linked to *in vitro* BBB permeability, such as MW below 450 – 500 Da, TPSA values lower than 90 Å^2^, and log*P*-values between 0 and 2, were investigated. Phenolic acids, flavonols, and methylated flavonoids showed high diffusion capacity in the *in vitro* BBB model, and hence are suggested as major bioactive compounds with neuroprotective activity, in line with previous research works. On the other hand, the presence of sugar moieties in the molecular structure was shown to impair the BBB permeability and the neuroprotective properties of the bioactive compounds.

The comprehensive chemical characterization followed by extensive *in vitro* bioactivity assessment of the present biomass extracts represents a step forward in the valorization of natural matrices as promising sources of neuroprotective compounds. Further *in vivo* model experiments are needed to understand the mechanism underlying the neuroprotective properties of these bioactive-rich extracts as promising sources of new functional foods and nutraceuticals with health-promoting properties against AD.

## Data Availability Statement

The original contributions presented in the study are included in the article/supplementary material, further inquiries can be directed to the corresponding author.

## Author Contributions

Conceptualization: JS-M, GA-R, AV, EI, and AC. Methodology: MA, RG, AV, ZS-M, and JS-M. Formal analysis and data curation: JS-M, RG, and GA-R. Resources, supervision, and project administration: EI and AC. Writing and original draft preparation: JS-M and AV. Writing, reviewing, and editing: GA-R, EI, and AC. Visualization: JS-M and GA-R. Funding acquisition: AC. All authors have read and agreed to the published version of the manuscript.

## Funding

This work was supported by the Ministry of Economy and Competitiveness (project PID2020-113050RB-I00).

## Conflict of Interest

The authors declare that the research was conducted in the absence of any commercial or financial relationships that could be construed as a potential conflict of interest.

## Publisher's Note

All claims expressed in this article are solely those of the authors and do not necessarily represent those of their affiliated organizations, or those of the publisher, the editors and the reviewers. Any product that may be evaluated in this article, or claim that may be made by its manufacturer, is not guaranteed or endorsed by the publisher.
